# Improved protein surface comparison and application to low-resolution protein structure data

**DOI:** 10.1186/1471-2105-11-S11-S2

**Published:** 2010-12-14

**Authors:** Lee Sael, Daisuke Kihara

**Affiliations:** 1 Department of Computer Science, Purdue University, 305 North University Street, West Lafayette, IN, 47907, USA; 2 Department of Biological Sciences, Purdue University, Hockmyer Hall of Structural Biology, 249 S. Martin Jischke Drive, West Lafayette, IN, 47907, USA; 3 Markey Center for Structural Biology, Purdue University, Hockmyer Hall of Structural Biology, 249 S. Martin Jischke Drive, West Lafayette, IN, 47907, USA

## Abstract

**Background:**

Recent advancements of experimental techniques for determining protein tertiary structures raise significant challenges for protein bioinformatics. With the number of known structures of unknown function expanding at a rapid pace, an urgent task is to provide reliable clues to their biological function on a large scale. Conventional approaches for structure comparison are not suitable for a real-time database search due to their slow speed. Moreover, a new challenge has arisen from recent techniques such as electron microscopy (EM), which provide low-resolution structure data. Previously, we have introduced a method for protein surface shape representation using the 3D Zernike descriptors (3DZDs). The 3DZD enables fast structure database searches, taking advantage of its rotation invariance and compact representation. The search results of protein surface represented with the 3DZD has showngood agreement with the existing structure classifications, but some discrepancies were also observed.

**Results:**

The three new surface representations of backbone atoms, originally devised all-atom-surface representation, and the combination of all-atom surface with the backbone representation are examined. All representations are encoded with the 3DZD. Also, we have investigated the applicability of the 3DZD for searching protein EM density maps of varying resolutions. The surface representations are evaluated on structure retrieval using two existing classifications, SCOP and the CE-based classification.

**Conclusions:**

Overall, the 3DZDs representing backbone atoms show better retrieval performance than the original all-atom surface representation. The performance further improved when the two representations are combined. Moreover, we observed that the 3DZD is also powerful in comparing low-resolution structures obtained by electron microscopy.

## Background

The number of protein structures deposited to the Protein Data Bank (PDB) is increasing at a rapid pace. Particularly, more and more protein structures of unknown function are being solved by structural genomics projects [1,2]. The flood of structures raises significant challenges to develop computational methods that will provide critical information for hypothesizing function and evolution of the proteins from their structural information [3]. The study of protein structures has now entered the informatics era, just as biological sequence analyses has done in the previous decade when efficient reuse of knowledge from existing databases became crucial. Speed is an essence in such analyses, since biologists would need to run various database searches using different tools, and such analyses would be conveniently performed if they are done in real-time. Most of the current structure comparison methods [4], such as those that compare main-chain orientations or corresponding residue positions[5,6], are designed for pair wise comparison and are not suitable for a fast database search.

There are other new tasks which have been brought up by recent experimental techniques, such as electron microscopy (EM), which provides low-resolution structure data. Here, challenges include how to use a low-resolution EM density map for fitting high resolution structures [7-9] or guiding protein structure prediction [10], and how to efficiently and accurately compare global and local structures [11,12]. Thus, development of a new generation of structure analysis tools, which allow a fast screening of large structure databases and can handle low resolution structure data, is needed. With this in mind, we represent protein structures as surface shapes by the 3D Zernike descriptors (3DZD) [13-17]. There have been previous works which employ a protein surface representation [15], such as volumetric representation [18], convex hull [19], and the spin image [20]. Compared to those works, the 3DZD has the following properties that make it ideal for use in protein shape analyses. First, it is rotation and translation invariant. Thus, prior time-consuming structure alignment is not necessary for structure comparison since their position in space does not change their 3DZDs. This property enables direct comparison of the EM density maps where atomic coordinates are not available. Another advantage is its compact representation; a 3D shape is effectively represented in only 121 (when the order is 20) numbers called the invariants. The 3DZD can also represent physicochemical properties such as the electrostatic potential and the hydrophobicity on the protein surfaces [16]. Recently the 3DZD has been further applied for protein-protein docking prediction [21], local surface comparison [22], pocket shape matching for structure-based function prediction [23], and ligand molecule screening [13,24].

In the previous work, we showed that the all atom surface representation by the 3DZD agrees sufficiently with the main-chain based structure comparison by CE [6], but some differences were also observed due to the variations in the representation [14]. The new contribution of this paper is two fold. First, we introduce three main-chain atom based surface representations which are found to better agree with the two existing structure classifications, the CE and the SCOP database [25], as compared with the previous all-atom surface representation. Second, we show that the proposed representation also allows for a fast and accurate database search for EM density maps.

## Methods

### Datasets

To examine structure retrieval performance of the proposed methods, we use a data set of 2337 representative protein structures, which are arbitrarily selected from 185 fold groups defined in a protein classification database by CE (ftp://ftp.sdsc.edu/pub/sdsc/biology/CE/db/ata_3.8_jul-2004.txt.gz). CE is one of the frequently used protein structure comparison programs that compares Cα positions of proteins using a dynamic programming algorithm. These representative structures have a resolution of 3.0 Å or better, have no more than 10 missing residues in the solved structure, have all heavy atom positions solved, and are longer than 100 residues. In addition, the structure similarity of each pair is less than a Z-score of 3.8 by CE.

This dataset also provides the SCOP classification code of proteins, which classifies the proteins into 8 class groups, 149 folds groups, 187 superfamily groups, and 279 family groups. We use both CE-based and SCOP classifications in our study since they have the following complementary features: The CE classification is automatic without human intervention and considers main-chain orientation, while SCOP is curated manually at a certain degree to take evolution into account.

At this juncture, it is important to note that there is no golden standard in classification of proteins. The structure similarity measured for different representations can be largely different for distantly related proteins since they capture different aspects of the structures [4]. In our previous paper [14], we showed that CE and SCOP do not fully agree and also that DALI [26], which compares distance maps of proteins, and CE have poorer agreement than CE and the 3DZD. Each method has its own strength and thus an appropriate method should be selected depending on the purpose of each study. We have further shown examples of proteins whose surface shape similarity infers functional similarity, which are not detected by the conventional sequence or main-chain structure comparison methods [14]. In this study, we demonstrate that the new main-chain surface representations encoded by the 3DZD have a better agreement to CE and SCOP as compared to the original all-atom surface representation introduced in the previous study [16].

### Computing protein surfaces

For a protein structure, four different surface representations are computed: one that uses all heavy atoms (AASurf), the backbone conformation with all heavy atoms in the main-chain, *i.e.* Cα, C, N, and O atoms (CACNO), the backbone with Cα, C, and N atoms (CACN), and the backbone Cα atoms only (CA). For the set of extracted atoms, the surface is generated using the MSMS program [27]. MSMS rolls a probe sphere on the atoms and defines the surface as the path of the center of the probe. The radius of the probe sphere is set to default value of 1.5Å for AASurf, CACNO, and CACN and the radius is set to 2.0Å for CA to generate a smoother representation. The generated surface is then mapped on a 3D grid. A grid cell (voxel) is assigned a value of 1 if it is on the surface and 0 otherwise. Because the 3DZD is defined within a unit sphere, the protein surfaces represented by voxels are scaled into a unit sphere. Therefore, the size information of the protein is lost. The resulting voxels are considered as an input 3D function, *f(x)*, which is used as input for computing the 3DZD as described in the next section.

### 3D Zernike descriptors

The 3DZD is a series expansion of an input 3D function, which allows for a compact representation of the 3D object (*i.e.* the input 3D function) [17,28]. The mathematical foundation of the 3DZD was laid out by Canterakis (1999) and was applied on 3D object retrieval by Novotni and Klein (2003). For readers’ convenience, a brief mathematical derivation of the 3DZD is shown below. For detailed derivations and discussions, refer to the aforementioned two papers Canterakis [29] and Novotni and Klein [30].

The first step of computing the 3DZD is derivation of the 3D Zernike moments. For an input 3D function, *f(x)*, the 3D Zernike polynomials defined on order *n*, degree *l*, and repetition *m*, are given by(1)

subjected to *-l < m < l , 0 ≤ l ≤ n* , and *(n-l)* being even. The spherical harmonics, , are functions of a set of a polar angle, ϑ, and a azimuthal angle, *ϕ*. The radial function, *R_nl_(r)*, incorporates the radius information into the basis function and are constructed so that  are polynomials when written in terms of the Cartesian coordinates. The 3D Zernike moments of *f(x)* are defined as the coefficients of the expansion using this orthonormal basis in the following formula:(2)

After computing the 3D Zernike moments, a normalization step is necessary to obtain rotation invariance. This is done by taking the L2 norm of the 3D Zernike moments as the descriptor. That is, the moments are collected into (2*l*+1) dimensional vectors  and the rotational invariance is obtained by defining 3DZD, *F_nl_* , as the norm of vectors Ω*_nl_* :(3)

**Figure 1:**
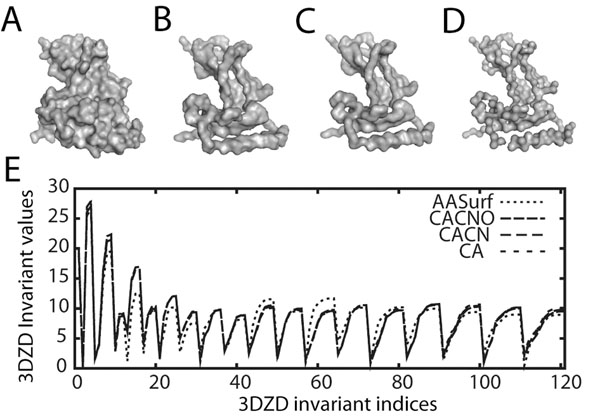
**Examples of four different representations of the protein (1hdmA).** A is surface representation using all-atoms (AASurf). B is a backbone representation using all heavy atoms in the main-chain (CACNO). C and D are simplified backbone representations which are composed of the atoms Cα, C, and N (CACN) and only the Cα atoms, respectively. E shows the 3DZD invariant values of the four representations.

The size of the 3DZD vector is set by the parameter *n*, called the order, which determines the resolution of the descriptor. The 3DZD is a series of invariants (Eqn. 3) for each combination of *n* and *l*, where *n* ranges from 0 to the specified order. For example, *n* ranges from 0 to 20 for a 3DZD of an order 20. The order of *n*=20, which yields a total of 121 numbers, or invariants, is used in our study based on the success of the previous works [14,30]. The last step is to normalize the descriptor by the norm of the descriptor. This normalization is found to reduce dependency of the 3DZD on the number of voxels used to represent a protein [14]. Figures 1A through D show the surface generated from the four representations. Figure 1E shows the 3DZD of the four representations for the protein PDB:1hdmA. It can be seen that there is little difference in the 3DZD of CACNO, CACN, and CA as compared to the 3DZD of AASurf in this particular case. The correlation coefficients among the three backbone representations (CACNO, CACN, and CA) range between 0.997 to 0.999. The correlation coefficients between CACNO, CACN, and CA to AASurf are 0.934, 0.938, and 0.941, respectively. Although there is little difference between the four representations in this particular example, we will show later that the four representations make a difference in terms of overall database retrieval performance.

### Evaluating database retrieval performance

The database retrieval performance of the four surface representations is evaluated with precision-recall curves. The precision-recall curves are often confused with the receiver operator characteristic curves. Although these two curves are related, the precision-recall curve is considered to be a better measure when the dataset is skewed [31]. The number of proteins in a group in the dataset used varies from 3 to 180 and thus a precision-recall is used here. For each protein in the dataset, the rest of the proteins described with the 3DZD are sorted by the Euclidean distance (L2 norm) to the 3DZD of the query protein. Then, the precision and the recall values are computed at each distance threshold value. The precision is defined as the fraction of the retrieved proteins of the same group with the query among all proteins retrieved above the distance threshold. The recall is defined as the fraction of the retrieved proteins of the same group with the query among all the proteins in the same group. Finally, we calculate the average precision and recall for each distance threshold. The precision-recall curves of different representations are evaluated by the area under curve (AUC).

As employed in the previous work [14], we also apply pre-filtering of the proteins by their sequence length. For a query, a protein in the database is filtered out if it is longer than 135% or shorter than 65% to the length of the query protein. This is done because of the loss of the size of the proteins during the process of computing the 3DZD, since the proteins are scaled to fit into a unit sphere.

### Combining 3DZD of AASurf and CACNO

We also examine database retrieval with combinations of the 3DZDs of the AASurf and a backbone surface representation. Among the three backbone representations, we choose CACNO since no significant difference in performance was observed among the three (see Results). CACNO would also be a natural choice since it is the full heavy atom representation of protein backbone. For the AASurf and CACNO combination, the distances measured independently are linearly combined with weighting factors:(4)

where **y** and **x** are the two proteins compared and *i* is the index of 3DZD invariants of AASurf, *S*, and CACNO, *B. w_yS_* and *w_yB_* are weights for AASurf and CACNO of the query protein **y**, and *m1* and *m2* are the number of invariants in the 3DZD of AASurf and CACNO, respectively. In this study, the 3DZD of AASurf and CACNO is set to the same size, *i.e.* m1=m2=121. Eqn. 4 is asymmetric since the weights *w_yS_* and *w_yB_* depend on the query protein, **y**.

The weights for AASurf and CACNO for a query protein are determined by two characteristics of its protein shape: 1) the existence of a tail-like structure and 2) the sphericity. The tail is an elongated region in the structure which is longer than three amino acids locating further than two times of the radius of gyration (RG) of the protein from the center of the gravity. The radius of gyration is defined as follows:(5)

where *N* is the number of atoms in protein *x_j_*, *cog*, is center of gravity of protein *x_j_*, and *R* is the approximate radius of atoms in which 1.5Å is used [32].

The sphericity measures how compactly a protein structure fits to a sphere:(6)

where *RS(x)* is the radius of a sphere that has the same volume as the protein all-atom surface representation computed by the MSMS program. A larger value indicates that the protein is more spherical.

## Results and discussion

### Database retrieval performance

**Figure 2:**
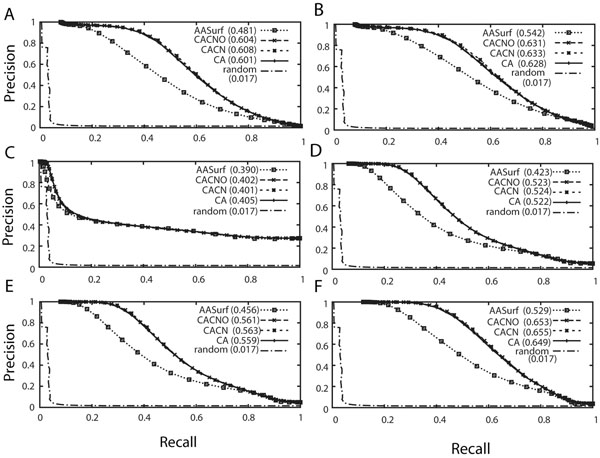
**Precision-recall curves using the CE and the SCOP classification.** The L2 norm is used to compute the distance between the two 3DZDs. A and B show precision-recall curves using the CE classification. In B, the length pre-filtering is applied. C, D, E and F are precision-recall graphs using the SCOP class, fold, superfamily, and family classification, respectively, as the base truth. The length pre-filtering is not used. The AUC values for the curves are shown inside brackets.

The database retrieval performance of the four surface representations is first tested on the CE classification. In all the precision-recall curves shown in Figure 2 (A-F), the four representations show significantly better performance than the random retrieval, which shows a precision-recall AUC value of 0.017. The random retrieval curve of the precision-recall graph is computed by generating random distance values between the pair of proteins. Readers should not be confused with the AUC value of the precision-recall used in this work with the one for the receiver operator characteristics (ROC) curves, which gives 0.5 for a random retrieval. Figure 2A and 2B show results without and with pre-filtering by protein length. All three backbone surface representations, CACNO, CACN, and CA, show similar performance, which is significantly better than that of AASurf. Note that AASurf showed much higher performance than DALI and four other shape-comparison methods in our previous work [14]. The length-based pre-filtering improves the AUC by around 0.03 for all the backbone representations and by 0.06 for AASurf representation. The length-based pre-filtering also improves the retrieval performance by incorporating the size information that is lost in the computation of 3DZD when a protein is scaled normalized. On the other hand, one could see that the improvement by the length filter is marginal, which implies it is not common to observe two proteins of different sizes with similar shape, which is consistent with the previous results [14].

Figure 2C, D, E, and F shows the retrieval performance based on the four hierarchical SCOP classifications: the class, the fold, the superfamily, and the family. The length-based pre-filtering was not applied. The improvement of the retrieval by the backbone representations over AASurf becomes more obvious as a more detailed level hierarchy is used, depicting that the backbone structure is more relevant in the family classification. In all the graphs in Figure 2 except for Figure 2C, CACN has the best retrieval performance followed by CACNO, CA, and AASurf. The reason of the better performance by CACNO, CACN, and CA over AASurf is that the former representations have more ruggedness, which can reflect the trace of the main-chain orientation better than AASurf. Using all atoms by AASurf makes many proteins look close to spheres or ellipsoids, and it is not advantageous in general to distinguish proteins of different folds.

Although CACN performs the best, the difference in performance by CACNO and CA is marginal with a average AUC difference of 0.003. Since the three backbone representations show almost identical performance, we will only show results of CACNO along with AASurf for further analyses.

### Comparison of CACNO and AASurf

**Figure 3:**
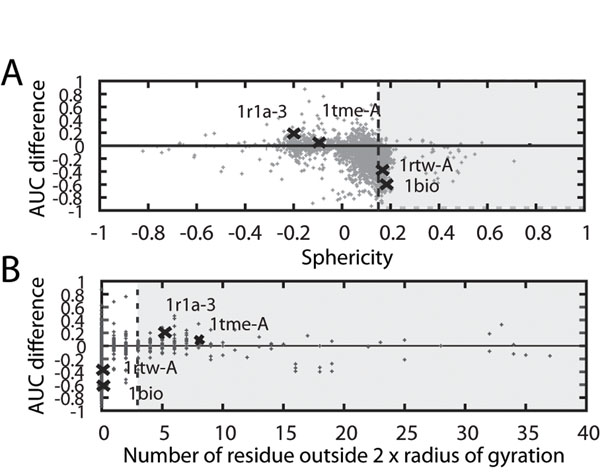
**Effect of the sphericity and the tail-like structure on the retrieval performance.** The effect of the sphericity and the tail-like structure to the precision-recall AUC of AASurf and CACNO representations is shown. A shows the effect of the sphericity, and B shows the effect of the number of the residues which reside further than two times of the radius of gyration (tail-like structures).

Close examination of the individual cases of the database retrieval revealed interesting trends on the performance of the AASurf and the CACNO representations. In Figure 3, the effect of the sphericity (Fig. 3A) and the tail-like structures (Fig. 3B) in the individual proteins are examined in respect of the database retrieval. Here, the CE classification is used as the base truth. The y-axis shows the difference of the AUC of the precision-recall curves with CACNO being subtracted from that of AASurf. Thus, a positive value indicates that the AASurf performs better than the CACNO and a negative value indicates the opposite.

Figure 3A indicates that CACNO tends to perform better than AASurf for spherical proteins (proteins with positive sphericity value). This is because CACNO gives more distinctive structural features which are characteristics for each group. For spherical proteins, AASurf shapes alone do not clearly separate proteins of the same group from spherical proteins from the other groups. On the other hand, AASurf performs better for proteins with a long tail structure (Fig. 3B). The 3DZD also tends to overestimate the similarity of the tail-like structures for proteins of different fold groups. A larger volume and structural information from the main body of the structure seems to help AASurf in recognizing the correct structure from the same group with the query.

**Figure 4:**
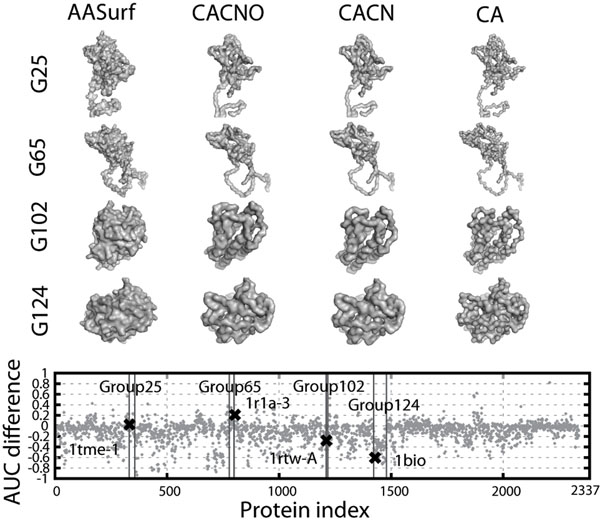
**Differences in database retrieval performance of AASurf and backbone representations**. The graph at the bottom shows the precision-recall AUC difference of AASurf to CACNO. The positive value indicates that the AASurf has a better performance. Four proteins are shown as examples. The first two, PDB:1tme1 in Group 25 (G25) and PDB:1r1a3 in G65 are examples where AASurf shows a better performance, while the latter two, PDB:1rtwA in G102 and PDB:1bio in G124 are cases where CACNO performs better. 1tme1 has the AUC difference of 0.0656, the sphericity (sp) of -0.104, and has 8 residues further than two times of the radius of gyration from the center. 1r1a3: AUC difference: 0.146, sp: -0.199, and 5 tail-like residues. 1rtwA, the AUC difference: -0.387, sp: 0.152, and no tail residues 1bio, the AUC difference: -0.597, sp: 0.165, and no tail residues.

Figure 4 shows the AUC difference with the AASurf and CACNO representations of individual proteins. On the x-axis, proteins are ordered in a way that proteins of the same classification are located next to each other. For many proteins, the two representations do not make much difference (51.8% of the proteins have a precision-recall AUC difference of less than 0.1), however, there are proteins for which the two representations show a large difference in the AUC value. Four examples are presented. The first two proteins, PDB:1tme1 in Group 25 and PDB:1r1a3 in Group 64, are cases where AASurf performs better, while CACNO performs better in the following two cases: PDB:1rtwA in Group 102 and PDB:1bio in Group 124. The data points for these four proteins are specified in Figures 3 and 4. Obviously the first two examples have long tails, whereas the latter two are spherical structure with no tails.

The observed difference by AASurf and CACNO for individual cases inspired us to combine the two representations to improve the retrieval performance. Those results are shown in the next section. For this combined representation, spherical proteins (those which have the sphericity value of larger than 0.15, the gray regions in Fig. 3A) and proteins with tail-like structures (those which have more than 3 residues in a tail, the gray regions in Fig. 3B) use different weights when combining the distance.

### Improvement by combining AASurf and CACNO representations

Next, we examine combining the distances defined by AASurf and CACNO using Eqn. 4. Query proteins with tail-like structures are given a higher AASurf weight (*w_yS_*) and ones with high sphericity are given a higher value for the CACNO weight. All others are given fixed weights for AASurf (0.4) and CACNO (0.6). With the threshold value of three residues for the tail-like structure and 0.15 for the sphericity, 383 and 150 structures in the dataset fall into the category of structures with tails and spherical structures, respectively. Table 1 shows the database retrieval results of the AASurf and CACNO combination (named Surf(ace)-Back(bone) representation) with different weight values. Again note that the random retrieval has the precision-recall AUC value of 0.017.

**Table 1:** Precision-recall AUC improvement using weighted distance.

Representation	Weights	AUC	Improvement ^a)^
AASurf	-	0.481	-
CACNO	-	0.604	-
Surf-Back	0.5/0.5	0.605	0.001 (0.124)
	0.4/0.6	0.617	0.013 (0.136)
	0..3/0.7	0.619	0.015 (0.138)
	0.2/0.8	0.612	0.008 (0.131)

Note that a random retrieval has a precision-recall AUC of 0.017.

a) Difference of Precision-recall AUC of Surf-Back to CACNO. Difference to AASurf is shown in the parentheses.

Among the different weight values tested, the weight combination 0.3 and 0.7 performed the best. In this combination, the weights for AASurf (*w_yS_*) and CACNO (*w_yB_*) are set to 0.7 and 0.3, respectively, for query proteins with a tail-like structure. On the other hand, if a query protein is spherical and has no tail-like structure, *w_yS_* is set to 0.3 and *w_yB_* is set to 0.7. Otherwise *w_yS_* and *w_yB_* are set to 0.4 and 0.6, respectively. Overall, the 0.3/0.7 weight combination results in an AUC increase of 0.015 and 0.138 when compared with the retrieval results using CACNO and AASurf, respectively. Out of 185 fold groups in the dataset, 116 groups show improvement by Surf-Back. There are 53 groups where CACNO shows a better performance and 16 groups where AASurf performs better than Surf-Back.

**Figure 5:**
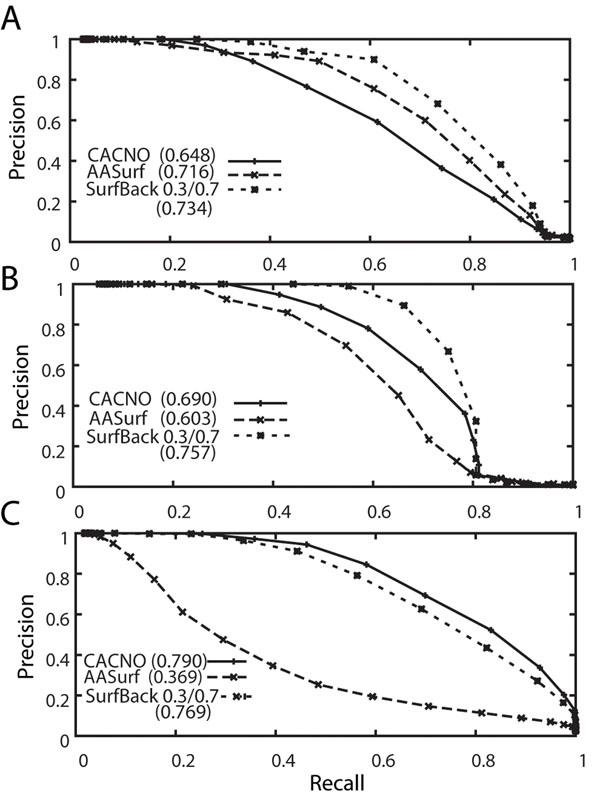
**Examples of precision-recall curves using the combination of AASurf and CACNO.** A, B, and C are precision-recall curves of Group 48, 74, and 124. AUC values for each curve are shown in brackets.

Figure 5 shows examples of precision-recall graphs of three fold groups. In the case of Group 48 (Fig. 5A), where AASurf performs better than CACNO, Surf-Back improves the AUC by 0.018 as compared to AASurf and improved by 0.086 as compared to CACNO. Group 74 (Fig. 5B) is an opposite example where CACNO performs better than AASurf. Surf-Back makes improvement by 0.154 and by 0.067 as compared to AASurf and CACNO, respectively. However, the linear combination of AASurf and CACNO does not always improve the retrieval accuracy. It cannot improve cases where one representation performs significantly worse than the other. The fold group 124 in Figure 5C shows such an example. Surf-Back performs significantly better than AAsurf but worse than the performance of CACNO by an AUC value of 0.021.

### Application to EM density maps

**Figure 6:**
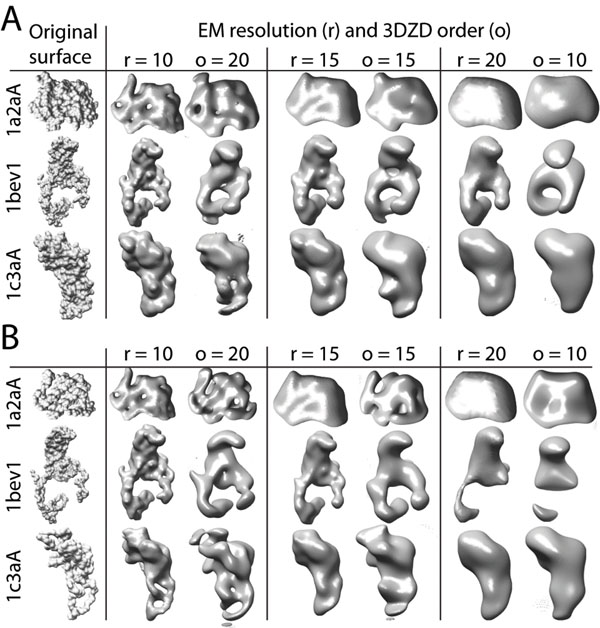
**Comparisons of isosurfaces of EM maps and reconstructed molecular surface from the 3DZD**. Three different EM resolutions (r=10, 15, and 20) and comparable surface shape reconstructed from the 3DZDs (order o = 20, 15, and 10) are shown. A, the 3DZDs are computed for AASurf representation of proteins, from which the surfaces are reconstructed. B, the CACNO representation is used to compute the 3DZDs.

Finally, we show that the 3DZD is readily applicable for comparing EM density maps. In Figure 6, low-resolution structures of isosurfaces of EM density maps and reconstructed structures of the 3DZDs (*i.e*. structural information coded in the 3DZDs) are visually compared. The EM density maps are generated with the pdb2mrc program which simulates EM density of protein structures [33]. The grid interval size is set to 1Å and three different resolutions (r=10, 15, and 20Å) are employed. In Fig. 6A, the original AASurf representations of proteins are reconstructed from their 3DZDs of three different orders (o=20, 15, and 10). The order which gives a similar resolution of the protein surface to each of the simulated EM density maps is chosen. For Figure 6B, 3DZDs of the CACNO representations are used for the reconstruction. Figure 6 shows that surface representation by 3DZD is visually similar to EM isosurfaces and thus would be suitable for describing EM density maps at different levels of resolutions.

Observing the agreement between the EM isosurface and structures coded by the 3DZD, we investigate further if the 3DZD can be used for searching similar EM density maps of protein structures (Table 2). To perform this experiment, we prepared datasets of the 3DZDs of EM density maps as follows: First, EM density maps of the 2337 representative protein structures are computed with pdb2mrc for two different resolutions, 10 and 15Å. Then, for an EM density map of a protein, two 3DZDs are computed, one using voxels with a high density value range (*e.g*. 9-11 for the resolution of 10) and another one using voxels with a low density value range (*e.g*. 5-8 for the resolution of 10; Table 2). Thus, a total of four datasets of 3DZDs of simulated EM isosurfaces are prepared. Voxels with a high density value in an EM density map locate at the core of a protein and thus its isosurface resembles the CACNO representation of the protein structure. On the other hand, the isosurface of a lower density value in the EM map looks more similar to the AASurf representation of the protein structure.

Table 2 summarizes the retrieval performance of the surfaces extracted from the simulated density maps described by the 3DZD. Here, the CE classification is used as base truth. To our surprise, the AUC values shown in Table 2 are as good as that of the AASurf representation of protein structures shown in Table 1 and Fig. 2A (0.481). Among those tested in Table 2, the 3DZDs of the order of 20 computed for EM density maps of 15Å resolution shows the highest AUC value, 0.489 (note again that this is much better than random retrieval). Indeed, this is higher than the result for the structural retrieval results with AASurf (Table 1). The results show that the EM density map of a relatively low resolution (15Å) can be as accurately compared as regular protein tertiary structures by using the 3DZD. Recent EM techniques can solve protein structures in much higher resolution, such as 4-6Å [34]. The significance of our results (Table 2) is that the 3DZD does not need such high resolutions for accurately comparing and searching EM density maps. Moreover, of course, the 3DZD should work better for EM maps with a higher resolution.

**Table 2:** Precision-recall AUC value of database retrieval of EM isosurfaces.

		AUC	
EM Resolution	Density Range ^a)^	3DZD order 15	3DZD order 20
10	5-8	0.427	0.451
	9-11	0.446	0.454
15	7-11	0.466	0.489
	12-15	0.460	0.480

a) Voxels with the specified density value range are chosen as input of computing 3DZD.

### Speed improvement of 3DZD over CE

A significant advantage of the 3DZD is its fast speed. Due to its compact vector representation and rotation invariance, 3DZD allows real-time search of the entire Protein Data Bank (PDB) [35] once 3DZDs of structures are computed offline and stored in the database. To illustrate, the speed by 3DZD is compared with CE in Table 3. This evaluation was performed on a computer with Intel core2 CPU 6400 (2.13GHz) processor with 5 Giga bytes memory. The pairwise structure comparison with 3DZD takes only 1.46x10^-4^s. Simply multiplying this execution time by the current size of PDB (64098 proteins) gives 9.36 seconds, while the same procedure by CE results will need almost 2 days. The speed of 3DZD is significantly faster than previous similar works on EM density map search [11,12].

**Table 3:** Computational speed.

	Steps	3DZD	CE
Preprocessing	Surface generation	1.42 (seconds)	-
	Voxelization	3.70	-
	Computing descriptor	7.83	-

Pair-wise comparison		1.46 x 10-4	2.66
PDB database scan ^a)^		9.36	1 day 23 hours 36 minutes

The values are averaged over the 2337 proteins. a) Times for the search against 64098 proteins.

## Conclusions

In this work we examined the applicability of the 3DZD for two important tasks in structural bioinformatics. The first task is the real-time protein structure database search. In contrast to our previous work [14] in which the 3DZD is used to represent an all-atom surface of protein structures (called in AASurf representation in this work), we have now examined backbone amino acid-based surface representations (*e.g.* CACNO). The backbone-based representations showed significantly better performance than the AASurf when the retrieval performance was evaluated on the agreement to the CE and SCOP classifications. Combinations of AASurf and CACNO showed further improvement over CACNO.

The second task explores the applicability of the 3DZD on representation and comparison of low-resolution structural data by evaluating the database retrieval performance of simulated EM density maps. Intuitively, isosurfaces of the density maps and molecular surfaces represented by 3DZDs look similar to each other. Indeed, we showed that the 3DZD is well suited for database retrieval of EM maps, achieving comparable accuracy to regular protein structure database retrieval in identifying proteins of the same fold to the query protein. This is the most comprehensive study so far published in identifying the fold class of proteins by comparing EM density map of proteins. It is noteworthy that the 3DZD can identify proteins of the same fold with EM maps even at 15Å resolution. Using EM maps of a higher resolution, which have now become more and more available, the retrieval accuracy will only get better. Here we compared the EM maps of single proteins as the proof of concept that 3DZD is suitable for handling EM maps. We expect this work will stimulate further investigations for applying 3DZD or similar descriptors for handling EM maps of multiple protein complexes and other low-resolution structure data, such as electron tomography.

Altogether, we are in a new exciting informatics era of structural biology, and we believe surface representation with the 3DZD will provide new directions and ideas that lead us to new findings through surfing ever expanding molecular structural information.

## List of abbreviations used

EM, electron microscopy; 3D, three dimensional; 3DZD, three dimensional Zernike descriptor; SCOP, structural classification of proteins (name of a database); CE, combinatorial extension (name of a protein structure comparison program); AASurf, all heavy atom surface; CA, alpha carbon; CACNO, a surface representation using C-alpha, C, N, O atoms in the main-chain; CACN, surface representation using C-alpha, C, N, atoms in the main-chain; MSMS, molecular surface calculation program by Michel Sanner; AUC, area under the curve; ROC, receiver operator characteristics; DALI, Distance matrices alignment algorithm; PDB, Protein Data Bank

## Competing interests

The authors declare that they have no competing interests.

## Authors' contributions

DK conceived the study and SL and DK designed the experiments. SL executed the experiments. SL drafted the manuscript and DK revised it critically. All authors read and approved the manuscript.
